# Chloridobis(ethane-1,2-diamine)(4-methyl­aniline)cobalt(III) dichloride monohydrate

**DOI:** 10.1107/S1600536809043323

**Published:** 2009-10-28

**Authors:** K. Ravichandran, P. Ramesh, C. Maharaja Mahalakshmi, K. Anbalagan, M. N. Ponnuswamy

**Affiliations:** a Centre of Advanced Study in Crystallography and Biophysics, University of Madras, Guindy Campus, Chennai 600 025, India; b Department of Chemistry, Pondicherry University, Pondicherry 605 014, India

## Abstract

In the title compound, [CoCl(C_2_H_8_N_2_)_2_(C_7_H_9_N)]Cl_2_·H_2_O, the Co^III^ ion has a distorted octa­hedral coordination environment and is surrounded by four N atoms in an equatorial plane, with the other N and Cl atoms occupying the axial positions. The crystal packing is stabilized by N—H⋯O, N—H⋯Cl and O—H⋯Cl inter­actions.

## Related literature

For the biological activity and potential applications of mixed ligand cobalt(III) complexes, see: Arslan *et al.* (2009 ▶); Delehanty *et al.* (2008 ▶); Sayed *et al.* (1992 ▶); Teicher *et al.* (1990 ▶). For Co—N and Co—Cl bond lengths in related complexes, see: Anbalagan *et al.* (2009 ▶); Lee *et al.* (2007 ▶); Ramesh *et al.* (2008 ▶); Ravichandran *et al.* (2009 ▶). For the preparation of dichloro­bis(1,2-diamino­ethane)cobalt(III) chloride, see: Bailer & Clapp (1945 ▶). For asymmetry parameters, see: Nardelli (1983 ▶). For puckering parameters, see: Cremer & Pople (1975 ▶).
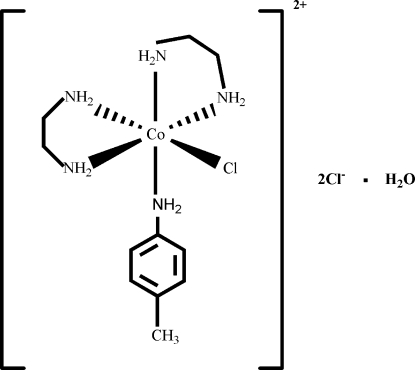


## Experimental

### 

#### Crystal data

[CoCl(C_2_H_8_N_2_)_2_(C_7_H_9_N)]Cl_2_·H_2_O
*M* *_r_* = 410.66
Triclinic, 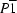

*a* = 7.3796 (2) Å
*b* = 10.8367 (3) Å
*c* = 12.1789 (3) Å
α = 75.201 (1)°
β = 74.671 (2)°
γ = 78.862 (1)°
*V* = 899.86 (4) Å^3^
*Z* = 2
Mo *K*α radiation
μ = 1.40 mm^−1^
*T* = 293 K
0.20 × 0.20 × 0.15 mm

#### Data collection

Bruker Kappa APEXII CCD diffractometer
Absorption correction: multi-scan (*SADABS*; Bruker, 2004 ▶) *T*_min_ = 0.767, *T*_max_ = 0.817
27923 measured reflections
8633 independent reflections
6851 reflections with *I* > 2σ(*I*)
*R*_int_ = 0.025

#### Refinement

*R*[*F* ^2^ > 2σ(*F* ^2^)] = 0.032
*wR*(*F* ^2^) = 0.091
*S* = 1.10
8633 reflections
239 parameters
H atoms treated by a mixture of independent and constrained refinement
Δρ_max_ = 0.50 e Å^−3^
Δρ_min_ = −0.33 e Å^−3^

### 

Data collection: *APEX2* (Bruker, 2004 ▶); cell refinement: *SAINT* (Bruker, 2004 ▶); data reduction: *SAINT*; program(s) used to solve structure: *SHELXS97* (Sheldrick, 2008 ▶); program(s) used to refine structure: *SHELXL97* (Sheldrick, 2008 ▶); molecular graphics: *ORTEP-3* (Farrugia, 1997 ▶); software used to prepare material for publication: *SHELXL97* and *PLATON* (Spek, 2009 ▶).

## Supplementary Material

Crystal structure: contains datablocks global, I. DOI: 10.1107/S1600536809043323/bt5082sup1.cif

Structure factors: contains datablocks I. DOI: 10.1107/S1600536809043323/bt5082Isup2.hkl

Additional supplementary materials:  crystallographic information; 3D view; checkCIF report

## Figures and Tables

**Table 1:** Hydrogen-bond geometry (Å, °)

*D*—H⋯*A*	*D*—H	H⋯*A*	*D*⋯*A*	*D*—H⋯*A*
N1—H1*B*⋯Cl2^i^	0.873 (17)	2.465 (17)	3.2553 (11)	150.8 (14)
N4—H4*B*⋯Cl2^ii^	0.797 (17)	2.602 (18)	3.3299 (11)	152.5 (15)
O1—H1*W*⋯Cl3^iii^	0.79 (3)	2.34 (3)	3.1339 (14)	174 (2)
O1—H2*W*⋯Cl3^iv^	0.77 (3)	2.49 (3)	3.2470 (14)	170 (2)
N9—H9*A*⋯Cl3	0.87 (2)	2.45 (2)	3.3152 (11)	168.9 (17)
N8—H8*B*⋯Cl2	0.82 (2)	2.57 (2)	3.3046 (12)	150.8 (18)
N9—H9*B*⋯O1	0.841 (17)	2.090 (17)	2.9250 (16)	172.1 (15)
N1—H1*A*⋯Cl3	0.88 (2)	2.39 (2)	3.2467 (11)	163.5 (17)
N4—H4*A*⋯O1	0.801 (17)	2.461 (17)	3.0754 (17)	134.3 (14)

Symmetry codes: (i) 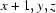; (ii) 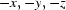; (iii) 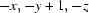; (iv) 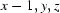.

**Fig. 1.**
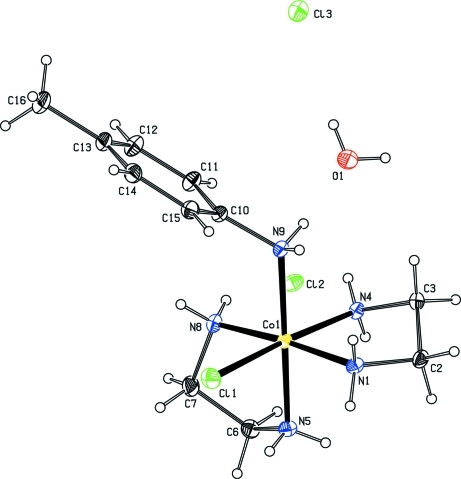
Perspective view of the molecule showing the displacement ellipsoids at the 30% probability level.

**Fig. 2.**
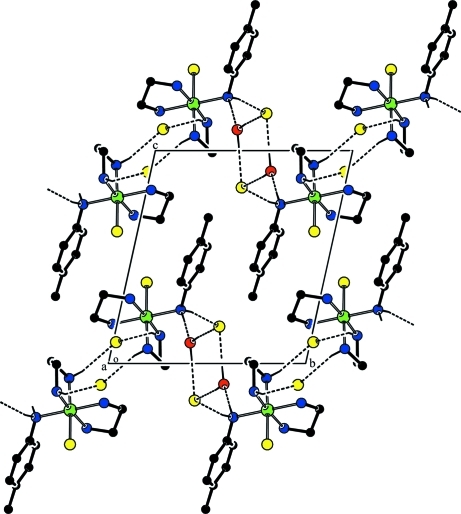
The crystal packing of the molecules viewed down the a–axis. H atoms not involved in hydrogen bonding have been omitted for clarity.

## supplementary crystallographic information

### Comment

Mixed ligand cobalt(III) complexes find potential applications in the fields of antitumor, antibacterial, antimicrobial, radiosenzitation and cytotoxicity activities (Sayed *et al.*,  1992; Teicher *et al.*, 1990; Arslan *et al.*,  2009; Delehanty *et al.*,  2008). Cobalt is an essential and integral component of vitamin B_12_, therefore it is physiologically found in most tissues. Complexes of cobalt are useful for nutritional supplementation to provide cobalt in a form which effectively increases the bioavailability, for instance, vitamin B_12_ by microorganisms present in the gut. In addition, cobalt(III) complexes are known for electron transfer and ligand substitution reactions, which find applications in chemical and biological systems. Against this background and to ascertain the molecular conformation, the structure determination of the title compound has been carried out.

The *ORTEP* diagram of the title compound is shown in Fig.1. The coordination geometry around the Co^III^ ion can be described as a slightly distorted octahedral. The Co^III^ ion and the four N atoms almost lie in the same plane, whereas the other N and Cl atoms are approximately perpendicular to this plane. The Co—N and Co—Cl bond lengths are comparable with related complexes (Lee *et al.*,  2007; Ramesh *et al.*, 2008; Anbalagan *et al.*,  2009; Ravichandran *et al.*,  2009). One of the five membered rings in the molecule adopts an envelope conformation, whereas the other ring adopts a twist conformation with the puckering parameters (Cremer & Pople, 1975) and the asymmetry parameters (Nardelli, 1983) for the ring Co1/N1/C2/C3/N4 are: q_2_ = 0.430 (1) Å, φ= 100.4 (1)° and Δ_2_(Co1)= 9.92 (9)°; and for the ring Co1/N5/C6/C7/N8 are: q_2_ = 0.428 (2) Å, φ= 272.0 (2)° and Δ_2_(Co1)= 1.9 (1)°.

The crystal packing is controlled by N—H···O, N—H···Cl and O—H···Cl interactions.

### Experimental

The complex was synthesized using dichlorobis(1,2-diamino ethane) cobalt(III) chloride by the reported method (Bailer & Clapp, 1945). A paste was prepared in a mortar with 2 g of *trans*-[Co^III^(en)_2_Cl_2_]Cl crystals in 3–4 drops of deionized water. To the solid mass, about 2.5 g of 4-methyl aniline dissolved in ethanol was added in drops for 20 min. The grinding was continued for half an hour and the color was found to change from dull green to red. The reaction mixture was set aside until no further change was observed and the product was allowed to stand overnight. Finally, the solid was washed with 3–4 times using pure ethanol. The final complex was dissolved in 5–10 ml of deionized water pre-heated to 70°C. The cobalt(III) complex was recrystallized out on addition of few drops of hot conc. HCl and 2 ml of water and cooled. The crystals were filtered, washed with ethanol and dried over vacuum.

### Refinement

Nitrogen and Oxygen H atoms were freely refined. Other H atoms were positioned geometrically (C—H = 0.93–0.97 Å) and allowed to ride on their parent atoms, with 1.5*U*_eq_(C) for methyl H and 1.2 *U*_eq_(C) for other H atoms.

### Crystal data

**Table:**

[CoCl(C_2_H_8_N_2_)_2_(C_7_H_9_N)]Cl_2_·H_2_O	*Z* = 2
*M**_r_* = 410.66	*F*(000) = 428
Triclinic, *P*1	*D*_x_ = 1.516 Mg m^−^^3^
Hall symbol: -P 1	Mo *K*α radiation, λ = 0.71073 Å
*a* = 7.3796 (2) Å	Cell parameters from 5716 reflections
*b* = 10.8367 (3) Å	θ = 2.4–32.8°
*c* = 12.1789 (3) Å	µ = 1.40 mm^−^^1^
α = 75.201 (1)°	*T* = 293 K
β = 74.671 (2)°	Block, red
γ = 78.862 (1)°	0.20 × 0.20 × 0.15 mm
*V* = 899.86 (4) Å^3^	

### Data collection

**Table:**

Bruker Kappa APEXII CCD diffractometer	8633 independent reflections
Radiation source: fine-focus sealed tube	6851 reflections with *I* > 2σ(*I*)
graphite	*R*_int_ = 0.025
ω and φ scans	θ_max_ = 37.6°, θ_min_ = 1.8°
Absorption correction: multi-scan (*SADABS*; Bruker, 2004)	*h* = −11→11
*T*_min_ = 0.767, *T*_max_ = 0.817	*k* = −18→18
27923 measured reflections	*l* = −20→20

### Refinement

**Table:**

Refinement on *F*^2^	Primary atom site location: structure-invariant direct methods
Least-squares matrix: full	Secondary atom site location: difference Fourier map
*R*[*F*^2^ > 2σ(*F*^2^)] = 0.032	Hydrogen site location: inferred from neighbouring sites
*wR*(*F*^2^) = 0.091	H atoms treated by a mixture of independent and constrained refinement
*S* = 1.10	*w* = 1/[σ^2^(*F*_o_^2^) + (0.0444*P*)^2^ + 0.0729*P*] where *P* = (*F*_o_^2^ + 2*F*_c_^2^)/3
8633 reflections	(Δ/σ)_max_ < 0.001
239 parameters	Δρ_max_ = 0.50 e Å^−^^3^
0 restraints	Δρ_min_ = −0.32 e Å^−^^3^

### Special details

**Table:**

Geometry. All e.s.d.'s (except the e.s.d. in the dihedral angle between two l.s. planes) are estimated using the full covariance matrix. The cell e.s.d.'s are taken into account individually in the estimation of e.s.d.'s in distances, angles and torsion angles; correlations between e.s.d.'s in cell parameters are only used when they are defined by crystal symmetry. An approximate (isotropic) treatment of cell e.s.d.'s is used for estimating e.s.d.'s involving l.s. planes.
Refinement. Refinement of *F*^2^ against ALL reflections. The weighted *R*-factor *wR* and goodness of fit *S* are based on *F*^2^, conventional *R*-factors *R* are based on *F*, with *F* set to zero for negative *F*^2^. The threshold expression of *F*^2^ > σ(*F*^2^) is used only for calculating *R*-factors(gt) *etc*. and is not relevant to the choice of reflections for refinement. *R*-factors based on *F*^2^ are statistically about twice as large as those based on *F*, and *R*- factors based on ALL data will be even larger.

### Fractional atomic coordinates and isotropic or equivalent isotropic displacement parameters (Å^2^)

**Table:**

	*x*	*y*	*z*	*U*_iso_*/*U*_eq_	
Cl1	0.22560 (5)	0.10374 (4)	0.37990 (3)	0.03929 (8)	
Cl2	−0.29764 (4)	0.01499 (3)	0.10024 (3)	0.03307 (7)	
Cl3	0.25518 (5)	0.52141 (3)	0.17368 (3)	0.03977 (8)	
Co1	0.11254 (2)	0.140646 (14)	0.218880 (12)	0.02132 (4)	
O1	−0.30863 (18)	0.39142 (13)	0.09756 (12)	0.0472 (3)	
H1W	−0.287 (3)	0.415 (2)	0.029 (2)	0.061 (7)*	
H2W	−0.408 (4)	0.424 (2)	0.122 (2)	0.061 (7)*	
N1	0.32886 (14)	0.23025 (10)	0.13118 (9)	0.02693 (18)	
H1A	0.319 (3)	0.3017 (19)	0.1556 (17)	0.047 (5)*	
H1B	0.435 (2)	0.1842 (16)	0.1437 (14)	0.031 (4)*	
C2	0.33739 (18)	0.26181 (12)	0.00430 (11)	0.0304 (2)	
H2A	0.4046	0.1897	−0.0302	0.036*	
H2B	0.4031	0.3362	−0.0337	0.036*	
C3	0.13641 (18)	0.29018 (12)	−0.01003 (10)	0.0305 (2)	
H3A	0.0761	0.3714	0.0109	0.037*	
H3B	0.1334	0.2954	−0.0902	0.037*	
N4	0.03717 (15)	0.18257 (11)	0.06885 (8)	0.02559 (18)	
H4A	−0.075 (3)	0.1988 (15)	0.0745 (14)	0.028 (4)*	
H4B	0.065 (2)	0.1235 (17)	0.0370 (15)	0.033 (4)*	
N5	0.25627 (16)	−0.02271 (11)	0.18526 (11)	0.0299 (2)	
H5A	0.325 (3)	−0.043 (2)	0.2275 (18)	0.051 (6)*	
H5B	0.319 (3)	−0.0173 (19)	0.1170 (18)	0.045 (5)*	
C6	0.1287 (2)	−0.12166 (12)	0.21421 (12)	0.0336 (2)	
H6A	0.1999	−0.2072	0.2270	0.040*	
H6B	0.0666	−0.1125	0.1511	0.040*	
C7	−0.0154 (2)	−0.10131 (13)	0.32353 (13)	0.0383 (3)	
H7A	−0.1160	−0.1536	0.3392	0.046*	
H7B	0.0437	−0.1251	0.3896	0.046*	
N8	−0.09254 (16)	0.03689 (11)	0.30385 (10)	0.02891 (19)	
H8A	−0.146 (3)	0.0534 (18)	0.3710 (18)	0.047 (5)*	
H8B	−0.177 (3)	0.0465 (18)	0.2693 (17)	0.043 (5)*	
N9	−0.04520 (15)	0.30641 (10)	0.25264 (9)	0.02624 (18)	
H9A	0.032 (3)	0.3621 (19)	0.2425 (17)	0.045 (5)*	
H9B	−0.113 (2)	0.3357 (15)	0.2038 (14)	0.028 (4)*	
C10	−0.17753 (17)	0.31173 (11)	0.36291 (10)	0.0269 (2)	
C11	−0.35600 (19)	0.27735 (14)	0.38350 (12)	0.0352 (3)	
H11	−0.3898	0.2479	0.3273	0.042*	
C12	−0.4850 (2)	0.28674 (16)	0.48792 (13)	0.0411 (3)	
H12	−0.6044	0.2621	0.5017	0.049*	
C13	−0.4384 (2)	0.33237 (14)	0.57219 (11)	0.0382 (3)	
C14	−0.2609 (2)	0.36910 (13)	0.54924 (11)	0.0370 (3)	
H14	−0.2289	0.4018	0.6041	0.044*	
C15	−0.12911 (19)	0.35829 (13)	0.44591 (11)	0.0320 (2)	
H15	−0.0093	0.3821	0.4324	0.038*	
C16	−0.5802 (3)	0.34315 (19)	0.68492 (14)	0.0541 (4)	
H16A	−0.5368	0.3933	0.7250	0.081*	
H16B	−0.5937	0.2587	0.7330	0.081*	
H16C	−0.7005	0.3844	0.6686	0.081*	

### Atomic displacement parameters (Å^2^)

**Table:**

	*U*^11^	*U*^22^	*U*^33^	*U*^12^	*U*^13^	*U*^23^
Cl1	0.04434 (18)	0.04594 (18)	0.03245 (15)	−0.00318 (14)	−0.01775 (13)	−0.00983 (13)
Cl2	0.02439 (13)	0.03889 (16)	0.03991 (15)	−0.00327 (11)	−0.00643 (11)	−0.01715 (13)
Cl3	0.04419 (18)	0.03590 (16)	0.04088 (17)	−0.01063 (13)	−0.00336 (13)	−0.01388 (13)
Co1	0.02056 (7)	0.02343 (7)	0.02069 (7)	−0.00416 (5)	−0.00377 (5)	−0.00610 (5)
O1	0.0374 (6)	0.0573 (7)	0.0410 (6)	0.0044 (5)	−0.0122 (5)	−0.0047 (5)
N1	0.0222 (4)	0.0276 (5)	0.0325 (5)	−0.0052 (4)	−0.0036 (4)	−0.0106 (4)
C2	0.0274 (5)	0.0302 (6)	0.0301 (5)	−0.0087 (4)	0.0021 (4)	−0.0057 (4)
C3	0.0310 (6)	0.0320 (6)	0.0246 (5)	−0.0060 (5)	−0.0022 (4)	−0.0018 (4)
N4	0.0240 (4)	0.0301 (5)	0.0236 (4)	−0.0064 (4)	−0.0034 (3)	−0.0075 (4)
N5	0.0272 (5)	0.0278 (5)	0.0347 (5)	−0.0025 (4)	−0.0055 (4)	−0.0094 (4)
C6	0.0392 (7)	0.0252 (5)	0.0365 (6)	−0.0066 (5)	−0.0068 (5)	−0.0068 (5)
C7	0.0456 (7)	0.0292 (6)	0.0343 (6)	−0.0101 (5)	−0.0008 (5)	−0.0012 (5)
N8	0.0285 (5)	0.0319 (5)	0.0253 (4)	−0.0084 (4)	−0.0011 (4)	−0.0064 (4)
N9	0.0258 (4)	0.0286 (5)	0.0235 (4)	−0.0034 (4)	−0.0022 (3)	−0.0078 (4)
C10	0.0276 (5)	0.0274 (5)	0.0245 (5)	−0.0015 (4)	−0.0029 (4)	−0.0081 (4)
C11	0.0319 (6)	0.0441 (7)	0.0317 (6)	−0.0065 (5)	−0.0021 (5)	−0.0161 (5)
C12	0.0330 (6)	0.0486 (8)	0.0384 (7)	−0.0093 (6)	0.0045 (5)	−0.0139 (6)
C13	0.0453 (8)	0.0352 (7)	0.0258 (5)	0.0013 (6)	0.0020 (5)	−0.0075 (5)
C14	0.0499 (8)	0.0350 (6)	0.0262 (5)	0.0001 (6)	−0.0087 (5)	−0.0111 (5)
C15	0.0355 (6)	0.0333 (6)	0.0295 (5)	−0.0036 (5)	−0.0085 (5)	−0.0104 (5)
C16	0.0601 (10)	0.0575 (10)	0.0318 (7)	0.0018 (8)	0.0083 (7)	−0.0135 (7)

### Geometric parameters (Å, °)

**Table:**

Cl1—Co1	2.2444 (3)	C6—H6B	0.9700
Co1—N1	1.9493 (10)	C7—N8	1.4777 (18)
Co1—N5	1.9600 (11)	C7—H7A	0.9700
Co1—N8	1.9648 (11)	C7—H7B	0.9700
Co1—N4	1.9673 (10)	N8—H8A	0.86 (2)
Co1—N9	2.0167 (10)	N8—H8B	0.82 (2)
O1—H1W	0.79 (3)	N9—C10	1.4439 (15)
O1—H2W	0.77 (3)	N9—H9A	0.87 (2)
N1—C2	1.4818 (17)	N9—H9B	0.841 (17)
N1—H1A	0.88 (2)	C10—C11	1.3797 (18)
N1—H1B	0.873 (17)	C10—C15	1.3876 (16)
C2—C3	1.5027 (18)	C11—C12	1.3866 (19)
C2—H2A	0.9700	C11—H11	0.9300
C2—H2B	0.9700	C12—C13	1.388 (2)
C3—N4	1.4849 (16)	C12—H12	0.9300
C3—H3A	0.9700	C13—C14	1.379 (2)
C3—H3B	0.9700	C13—C16	1.506 (2)
N4—H4A	0.801 (17)	C14—C15	1.3883 (19)
N4—H4B	0.797 (17)	C14—H14	0.9300
N5—C6	1.4768 (17)	C15—H15	0.9300
N5—H5A	0.78 (2)	C16—H16A	0.9600
N5—H5B	0.83 (2)	C16—H16B	0.9600
C6—C7	1.505 (2)	C16—H16C	0.9600
C6—H6A	0.9700		
			
N1—Co1—N5	90.39 (5)	N5—C6—H6A	110.4
N1—Co1—N8	175.15 (5)	C7—C6—H6A	110.4
N5—Co1—N8	84.75 (5)	N5—C6—H6B	110.4
N1—Co1—N4	84.77 (4)	C7—C6—H6B	110.4
N5—Co1—N4	90.54 (5)	H6A—C6—H6B	108.6
N8—Co1—N4	95.17 (4)	N8—C7—C6	106.93 (11)
N1—Co1—N9	91.51 (4)	N8—C7—H7A	110.3
N5—Co1—N9	177.58 (4)	C6—C7—H7A	110.3
N8—Co1—N9	93.34 (4)	N8—C7—H7B	110.3
N4—Co1—N9	88.15 (4)	C6—C7—H7B	110.3
N1—Co1—Cl1	89.09 (3)	H7A—C7—H7B	108.6
N5—Co1—Cl1	89.51 (4)	C7—N8—Co1	109.73 (9)
N8—Co1—Cl1	90.95 (4)	C7—N8—H8A	106.8 (13)
N4—Co1—Cl1	173.86 (3)	Co1—N8—H8A	112.7 (13)
N9—Co1—Cl1	92.01 (3)	C7—N8—H8B	107.1 (13)
H1W—O1—H2W	108 (2)	Co1—N8—H8B	113.5 (13)
C2—N1—Co1	111.40 (7)	H8A—N8—H8B	106.6 (18)
C2—N1—H1A	109.7 (13)	C10—N9—Co1	122.01 (8)
Co1—N1—H1A	108.8 (13)	C10—N9—H9A	106.4 (13)
C2—N1—H1B	108.9 (11)	Co1—N9—H9A	107.7 (13)
Co1—N1—H1B	111.2 (11)	C10—N9—H9B	103.3 (11)
H1A—N1—H1B	106.7 (17)	Co1—N9—H9B	109.0 (11)
N1—C2—C3	107.07 (10)	H9A—N9—H9B	107.5 (16)
N1—C2—H2A	110.3	C11—C10—C15	119.80 (11)
C3—C2—H2A	110.3	C11—C10—N9	120.15 (11)
N1—C2—H2B	110.3	C15—C10—N9	119.95 (11)
C3—C2—H2B	110.3	C10—C11—C12	120.01 (12)
H2A—C2—H2B	108.6	C10—C11—H11	120.0
N4—C3—C2	106.54 (10)	C12—C11—H11	120.0
N4—C3—H3A	110.4	C11—C12—C13	120.90 (14)
C2—C3—H3A	110.4	C11—C12—H12	119.6
N4—C3—H3B	110.4	C13—C12—H12	119.6
C2—C3—H3B	110.4	C14—C13—C12	118.40 (12)
H3A—C3—H3B	108.6	C14—C13—C16	121.16 (14)
C3—N4—Co1	108.95 (7)	C12—C13—C16	120.43 (15)
C3—N4—H4A	111.1 (12)	C13—C14—C15	121.37 (12)
Co1—N4—H4A	113.9 (11)	C13—C14—H14	119.3
C3—N4—H4B	107.5 (12)	C15—C14—H14	119.3
Co1—N4—H4B	112.7 (12)	C10—C15—C14	119.49 (13)
H4A—N4—H4B	102.4 (16)	C10—C15—H15	120.3
C6—N5—Co1	110.44 (8)	C14—C15—H15	120.3
C6—N5—H5A	108.2 (15)	C13—C16—H16A	109.5
Co1—N5—H5A	104.6 (15)	C13—C16—H16B	109.5
C6—N5—H5B	109.6 (13)	H16A—C16—H16B	109.5
Co1—N5—H5B	114.2 (13)	C13—C16—H16C	109.5
H5A—N5—H5B	110 (2)	H16A—C16—H16C	109.5
N5—C6—C7	106.72 (11)	H16B—C16—H16C	109.5
			
N5—Co1—N1—C2	82.07 (9)	N5—Co1—N8—C7	15.23 (9)
N8—Co1—N1—C2	81.1 (5)	N4—Co1—N8—C7	105.29 (9)
N4—Co1—N1—C2	−8.43 (8)	N9—Co1—N8—C7	−166.27 (9)
N9—Co1—N1—C2	−96.43 (8)	Cl1—Co1—N8—C7	−74.20 (9)
Cl1—Co1—N1—C2	171.58 (8)	N1—Co1—N9—C10	−142.93 (9)
Co1—N1—C2—C3	33.98 (12)	N5—Co1—N9—C10	75.2 (11)
N1—C2—C3—N4	−49.24 (13)	N8—Co1—N9—C10	37.28 (10)
C2—C3—N4—Co1	42.63 (11)	N4—Co1—N9—C10	132.35 (9)
N1—Co1—N4—C3	−19.47 (8)	Cl1—Co1—N9—C10	−53.79 (9)
N5—Co1—N4—C3	−109.82 (8)	Co1—N9—C10—C11	−81.78 (14)
N8—Co1—N4—C3	165.40 (8)	Co1—N9—C10—C15	101.80 (12)
N9—Co1—N4—C3	72.21 (8)	C15—C10—C11—C12	−1.4 (2)
Cl1—Co1—N4—C3	−19.4 (4)	N9—C10—C11—C12	−177.80 (13)
N1—Co1—N5—C6	−166.79 (9)	C10—C11—C12—C13	1.0 (2)
N8—Co1—N5—C6	13.13 (9)	C11—C12—C13—C14	0.5 (2)
N4—Co1—N5—C6	−82.01 (9)	C11—C12—C13—C16	179.51 (15)
N9—Co1—N5—C6	−24.9 (12)	C12—C13—C14—C15	−1.5 (2)
Cl1—Co1—N5—C6	104.13 (9)	C16—C13—C14—C15	179.43 (14)
Co1—N5—C6—C7	−37.90 (13)	C11—C10—C15—C14	0.34 (19)
N5—C6—C7—N8	50.01 (14)	N9—C10—C15—C14	176.77 (12)
C6—C7—N8—Co1	−39.65 (13)	C13—C14—C15—C10	1.1 (2)
N1—Co1—N8—C7	16.2 (6)		

### Hydrogen-bond geometry (Å, °)

**Table:**

*D*—H···*A*	*D*—H	H···*A*	*D*···*A*	*D*—H···*A*
N1—H1B···Cl2^i^	0.873 (17)	2.465 (17)	3.2553 (11)	150.8 (14)
N4—H4B···Cl2^ii^	0.797 (17)	2.602 (18)	3.3299 (11)	152.5 (15)
O1—H1W···Cl3^iii^	0.79 (3)	2.34 (3)	3.1339 (14)	174 (2)
O1—H2W···Cl3^iv^	0.77 (3)	2.49 (3)	3.2470 (14)	170 (2)
N9—H9A···Cl3	0.87 (2)	2.45 (2)	3.3152 (11)	168.9 (17)
N8—H8B···Cl2	0.82 (2)	2.57 (2)	3.3046 (12)	150.8 (18)
N9—H9B···O1	0.841 (17)	2.090 (17)	2.9250 (16)	172.1 (15)
N1—H1A···Cl3	0.88 (2)	2.39 (2)	3.2467 (11)	163.5 (17)
N4—H4A···O1	0.801 (17)	2.461 (17)	3.0754 (17)	134.3 (14)

Symmetry codes: (i) *x*+1, *y*, *z*; (ii) −*x*, −*y*, −*z*; (iii) −*x*, −*y*+1, −*z*; (iv) *x*−1, *y*, *z*.
